# Supplementary material for: Bigtools: a high-performance BigWig and BigBed library in Rust
Source: Bioinformatics. 2024 Jun 11;40(6):btae350. doi: 10.1093/bioinformatics/btae350 (PMC11167208; doi:10.1093/bioinformatics/btae350)
Supplement: btae350_Supplementary_Data [file btae350_supplementary_data.pdf]

Supplementary Information

Bigtools internal design considerations

BBI files generated by and read by Bigtools are fully compatible with the UCSC tools. However, Bigtools implements a number of design decisions that result in substantially better performance, both in memory and wall time.

BBI files contain an index referred to as a cirTree, which consists of an R tree to index primary data intervals, and a B+ tree to map chromosome names to integer IDs [Kent et al., 2010]. Whereas the UCSC tools write the B+ tree to disk before the primary data itself, Bigtools writes the B+ tree section between the primary data and the R tree index. This change allows Bigtools to eliminate an initial pass over input BED or bedGraph input files to collect chromosome usage information. While this pass requires relatively little computation, it is heavily I/O-bound and scales linearly with file size. This optimization alone is responsible for most of the reduction in wall time for writing compared to the UCSC tools. An alternative solution to avoid this initial pass would be to provide all chromosome information upfront but out-of-band (e.g., using a chromosome-sizes file). However, this solution introduces the tradeoff that unused chromosomes may be written to the output file as well.

The UCSC tools also make use of this first pass to (i) verify the file sort order and (ii) calculate the average size of the entries. The former can trivially be done during primary data writing, and the latter is not required if additional changes are made to zoom processing. Unlike UCSC tools, Bigtools can calculate zoom statistics on-the-fly during primary data processing. This is done by default on large files or when specified. There are two benefits to this approach. First, input data only needs to be read once, which also lets input be generated on the fly or streamed through `stdin`. Second, in multi-threaded scenarios, the compression of zoom data can be done in parallel with primary data processing.

While the UCSC tools use a starting zoom size of one quarter the average entry size, the biggest caveat to processing input files in a single pass is the requirement to determine the zoom sizes *a priori*. For this, Bigtools selects an initial zoom size of 160 base pairs by default. Both Bigtools and the UCSC tools calculate up to 10 zoom levels, but Bigtools filters zoom levels by size and section count before writing the final BBI file, such that bad estimates do not result in substantially larger file sizes. For moderate and large size files, this estimate results in similar zoom data as those produced by UCSC tools with little overhead from the extra processing of zoom levels that get filtered out. However, on very small files with large average entry size, considerable overhead goes into encoding zoom data that gets dropped. In these cases, selecting the multipass option (or if using as a library, configuring the initial zoom size) may be more appropriate.

During zoom level building, processed zoom data can be temporarily stored either in memory or in temporary files (the default) while the primary data is being written to disk. While the zoom data could, in theory, be interspersed with the primary data, we do not do this since, in practice, third party tools may read the primary data contiguously rather than relying on the index.

Architecturally, Bigtools uses Rust's `async` feature along with the `tokio` library to enqueue input file reading, section encoding, and BBI file writing as separate tasks. In a multi-threaded environment, this effectively means that data compression can be offloaded to separate threads, which alone accounts for about 85% of CPU time. Currently, Bigtools still relies at its core on blocking APIs to read input data which provides for a simpler user-facing API, but future refactorings may eventually allow full usage in non-blocking environments, such as WASM, or to better utilize networking for remote files.

Finally, when reading BBI files, Bigtools strives for laziness with respect to both memory usage and computation. For example, in `bigwigaverageoverbed`, Bigtools only reads one line of the input bed at a time, which not only reduces memory usage considerably, but saves considerable time since only a subset of the bigWig data must be read. Similarly, for `bigwigmerge`, Bigtools does not read all records for entire chromosomes into memory at once but instead loads and calculates merged data records incrementally. Furthermore, unlike its UCSC tools counterpart which writes to an intermediary bedGraph text file, Bigtools `bigwigmerge` can exploit the single-pass approach to write directly to a bigWig file while merging input data in parallel.

References

W. J. Kent, A. S. Zweig, G. Barber, A. S. Hinrichs, and D. Karolchik. BigWig and BigBed: enabling browsing of large distributed datasets. *Bioinformatics*, 26(17):2204–2207, 2010.
